# Conformal Partial Brain Irradiation Versus Stereotactic Radiation Therapy in the Management of Resected Brain Metastases: A Retrospective Study

**DOI:** 10.7759/cureus.77762

**Published:** 2025-01-21

**Authors:** Sophia N Shah, Sohan S Shah, Gaurav Shukla, Sunjay A Shah

**Affiliations:** 1 Radiation Oncology, Christiana Care Health System, Newark, USA

**Keywords:** 3d-conformal radiation therapy, leptomeningeal enhancement, stereotactic radiotherapy (srt), treatment of brain metastases, whole-brain radiotherapy

## Abstract

Introduction: The standard of care after resection of a single brain metastasis is to treat the cavity with stereotactic radiation therapy (SRT) to minimize the risk of recurrence. However, a prospective randomized trial of SRT demonstrated higher than expected rates of local recurrence, possibly due to geographic miss. Conformal partial brain (CPB) irradiation using conventional fractionation is an alternate technique that allows a larger margin of healthy tissue to be safely irradiated, potentially decreasing the risk of tumor recurrence. We performed a retrospective chart review to compare the results between CPB and SRT treatments.

Methods and materials: Patients receiving postoperative cranial radiotherapy within two months of a brain metastasis resection from 2015 to 2022 were eligible for this retrospective single-institution analysis. Fifty-seven patients met the eligibility criteria (SRT: n=32; CPB: n=25). SRT patients were treated using a robotic linear accelerator with a median dose of 24 Gy in 3 fractions. The median prescribed dose for the CPB group was 33 Gy in 11 fractions.

Results: The mean follow-up was 19.9 months. The crude rate of local recurrence rate was 21.9% (SRT) versus 0% (CPB) (p<0.013). The crude rate of radiation necrosis (RN) was 21.9% (SRT) versus 0% (CPB) (p<0.013). The mean cavity volume was 13 cc (SRT) versus 73 cc (CPB) (p<0.001). Most cases of RN were asymptomatic, although one patient suffered grade 4 status epilepticus.

Conclusion: In this single-institution cohort, CPB radiation therapy was statistically associated with a lower risk of both local failure and radiation necrosis as compared to SRT. Despite the cavity being much larger, none of the CPB patients suffered either local failure or radiation necrosis. Postoperative CPB irradiation may be beneficial for large cavity sizes or when it is difficult to delineate the tumor bed.

## Introduction

Brain metastases occur in 10% to 30% of all patients who are diagnosed with a systemic malignancy [1]. Historically, the presence of brain metastases has been a poor prognostic indicator, associated with a median survival of three to six months [2]. However, due to advances in systemic and local therapies, the life expectancy for cancer patients is increasing, with a resultant increase in the incidence of brain metastases. Traditionally, symptomatic metastases in patients with reasonable life expectancy have been removed by surgical resection. Because residual malignancy and local tumor spillage may be present after resection, surgery has historically been followed by whole-brain radiation therapy (WBRT) [3]. Patchell et al. reported that the local recurrence rate dropped from 52% to 20% when the surgical cavity was treated with adjuvant WBRT [4]. However, it has become increasingly recognized that WBRT can lead to significant permanent neurotoxicity, including short-term memory loss and decreased cognitive processing speed [5].

An alternative treatment strategy after metastatic resection is stereotactic radiosurgery (SRS) to the tumor bed. SRS is highly conformal and permits the preservation of brain tissue and delivery of a highly biologically effective dose (BED) to the tumor cavity. It may permit delay of the cognitive side effects of WBRT. SRS is ideal for treating well-defined targets such as intact metastases, but postoperative changes and the evolution of cavity size make it challenging to identify the volume at risk [6]. In addition, when surgical resection of brain metastases takes place, subclinical tumor seeding may occur along the tumor bed and surgical tract [7]. The combination of changing cavity size and subclinical seeding risks geographic miss and local recurrence. In a randomized trial by Brown et al., the local control (LC) rate with SRS in this trial was reported to be 40.7% at 12 months in the SRS group versus 81.5% at 12 months in the WBRT group (overall HR 3.12, p=0.0033) [8]. Nevertheless, SRS to the tumor bed was demonstrated to markedly increase the time to cognitive failure versus WBRT and has become the standard of care in the United States for patients with limited metastases [9]. Multiple fractions of radiosurgery, referred to as stereotactic radiation therapy (SRT), are frequently used for larger cavity volumes and are associated with reduced risk of radiation necrosis (RN) [10].

Another option with theoretical advantages is moderately dose-escalated conformal partial brain (CPB) radiation therapy. We hypothesized that the CPB would be a safer option for resected brain metastases due to more generous margins around the cavity, surgical tract, and dural surface, which should decrease the risk of a marginal miss. A safe, effective, and commonly utilized dose for WBRT is 3000 cGy in 10 fractions. However, partial brain volumes can safely be treated to a higher dose of 3300 cGy in 11 fractions with a low risk of radiation necrosis [11]. CPB at our institution was delivered with either 3-D conformal radiation therapy or intensity-modulated radiation therapy (IMRT), depending on the irregularity of the cavity volume and the presence of adjacent critical structures such as the brainstem, optic chiasm, and cochlea. We performed a retrospective chart review in our department to compare the results between CPB and SRT treatments.

## Materials and methods

We conducted a retrospective single-institution chart review of all patients treated with postoperative radiation SRT or CPB within two months of surgery between 2015 and 2022. Patients were treated in the tumor bed after brain metastasis resection with an SRT technique utilizing one to five fractions utilizing a robotic radiosurgery device or with an 11-fraction CPB technique with a conventional linear accelerator. The technique chosen was based on physician preference, with a greater likelihood of the CPB technique for large cavity sizes.

Patient selection

All patients underwent brain metastasis resection between September 2015 and January 2022, and subsequent treatment was given at a single community health care system. A search of the Mosaiq electronic medical records identified 26 patients treated with CPB and 39 patients who received SRT to the tumor bed within two months of brain metastasis resection. Patient demographics are described in Table 1.

**Table 1:** Comparison of patient characteristics between conformal partial brain (CPB) and stereotactic radiation therapy (SRT) treatment groups CPB: conformal partial brain; SRT: stereotactic radiation therapy

Characteristic	Overall sample N=55	CPB N=23 patients	SRT N=32
Age, mean (SD)	64.81 (12.29)	64.28 (16.27)	65.22 (8.21)
Male	28	10	18
Female	27	13	14

Patient treatments

**Figure 1:**
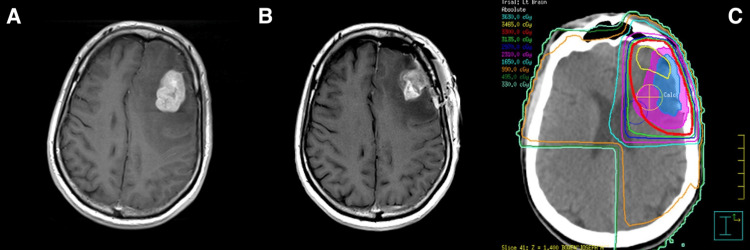
(A) MRI of brain metastasis prior to resection (B) MRI of tumor bed after resection and (C) 33 Gy/11 fractions dosimetry for a patient treated with conformal partial brain radiation

**Figure 2:**
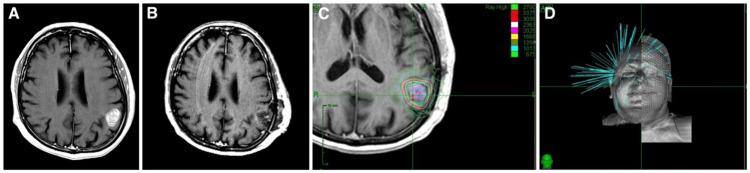
Examples of (A) MRI of brain metastasis prior to resection; (B) MRI of tumor bed after resection; (C) The 24 Gy/3 fraction dosimetry for stereotactic radiosurgery; and (D) CyberKnife beam arrangement

Prior to computed tomography (CT) simulation, patients were immobilized with a thermoplastic mask to limit patient movement. Pre- and post-surgery MRI images were transferred to the planning system and fused with the simulation CT scan for planning purposes. Target contours were reviewed during daily chart rounds before planning. A highly conformal treatment was designed using a RayStation treatment planning system for 3-D and IMRT plans. The CPB plans typically included the tumor bed, surgical tract, and dural surface with a 5 mm clinical target volume (CTV) margin and a 3 mm planning target volume (PTV) margin. 3-D plans were used unless it was felt that IMRT would better spare normal brain tissue due to irregular target geometry or proximity to critical structures (Figure 1). Robotic SRT treatments were planned on the Cyberknife Precision planning software (Accuray Incorporated, USA). Treatment was delivered using the CyberKnife skull tracking system. The SRT target volume typically included the tumor bed with a 3-5 mm CTV margin and no PTV margin (Figure 2).

Clinical endpoints

We evaluated the following clinical endpoints: time to local recurrence, time to distant recurrence, incidence of radiation necrosis, incidence of leptomeningeal disease, and overall survival. Local recurrence was defined as the radiographic presence of a recurrent tumor either within or adjacent to the previously treated tumor cavity or along the surgical margins. Pathologic confirmation was optional. Distant recurrence was defined as the occurrence of any intracranial metastasis not classified as a local recurrence. The time to local failure and distant brain failure was measured from the completion of treatment for brain metastases to the detection of disease on MRI. Radiation necrosis was established by multidisciplinary consensus after a radiological review. Perfusion MRIs were requested in cases that were inconclusive. Leptomeningeal failures were scored as classical versus nodular.

Statistical analysis

Mean (standard deviation) was used to present continuous variables, while frequency (percentage) was used for categorical variables. A statistician conducted the statistical analysis, and Kaplan-Meier curves were generated to evaluate time to local recurrence, distant recurrence, and survival time. Univariate and multivariate analyses were performed to identify predictors of postoperative radionecrosis. Statistical significance was defined as a p-value less than 0.05. All statistical analyses were carried out using R Studio, version 2023.03.0+386 (Posit PBC, Boston, United States). Variables evaluated for association with local failure included age, sex, Karnofsky performance status (KPS), extracranial metastases, more than one intracranial metastasis, histology, the volume of the cavity, the volume of PTV, the ratio of PTV/cavity, and the conformity index (CI).

Ethics approval

All of the procedures involving human subjects followed the ethical standards that were set by both national and institutional research committees. Christiana Care Institutional Review Board (IRB) granted an exception from ethical approval as it was a purely observational review (IRB # CCC42041 on June 14, 2022). The research was conducted according to the principles of the Declaration of Helsinki.

Informed consent

Because this study was a retrospective review using de-identified patient information, informed consent was not collected from the subjects.

## Results

Patient characteristics

Table 2 summarizes the patient cohort's characteristics. The median age at treatment was 65 years old, with a similar distribution of men and women (28 men and 27 women). The most prevalent histologies were non-small cell lung cancer at 47%, breast cancer at 14%, and renal cancer at 12%. Other histologies included melanoma, ovarian cancer, and small-cell lung cancer. The median cavity volume was 21.7 cc, with a standard deviation of 21.65 cc. A complete resection by MRI was achieved in 41 patients (71.95%).

**Table 2:** Comparison of brain metastasis characteristics between conformal partial brain (CPB) and stereotactic radiation therapy (SRT) treatment groups *p<0.001 t-test for difference in means: cavity size; t-test for difference in proportions: complete resection NSCLC: non-small cell lung cancer; SCLC: small cell lung cancer; CPB: conformal partial brain; SRT: stereotactic radiosurgery

Characteristic	Overall sample N=57	CPB N=25	SRT N=32	p-value
NSCLC, n (%)	27 (47.4%)	11 (44.0%)	16 (50.0%)	N/A
SCLC, n (%)	1 (1.8%)	0 (0.0%)	1 (3.1%)	N/A
Renal, n (%)	7 (12.3%)	3 (12.0%)	4 (12.5%)	N/A
Breast, n (%)	8 (14.0%)	5 (20.0%)	3 (9.4%)	N/A
Melanoma, n (%)	6 (10.5%)	3 (12.0%)	3 (9.4%)	N/A
Ovarian, n (%)	1 (1.8%)	0 (0.0%)	1 (3.1%)	N/A
Other/unknown, n (%)	7 (12.3%)	3 (12.0%)	4 (12.5%)	N/A
Cavity size, mean (SD)	39.4 (36.7)	73.2 (30.3)	13.0 (9.1)	<0.001*
Complete resection (yes), n (%)	41 (71.9%)	19 (76.0%)	22 (68.8%)	0.533

Clinical and treatment characteristics

Conformal Partial Brain

**Figure 3:**
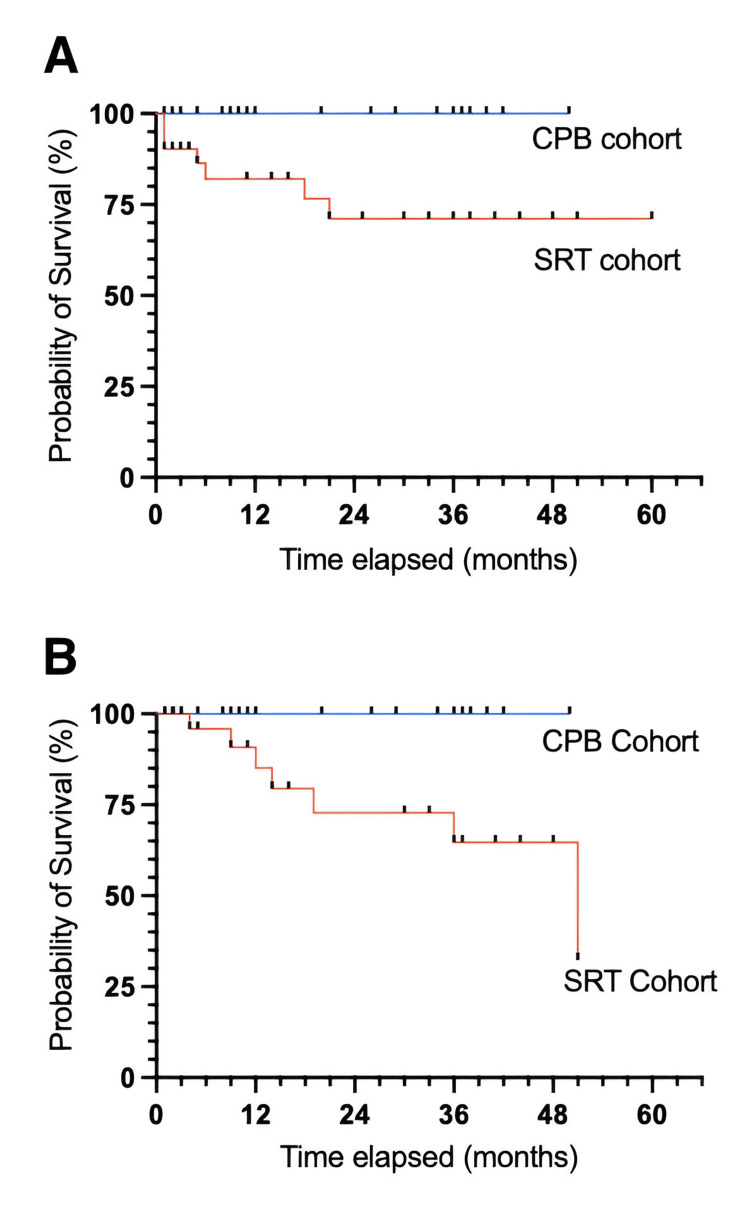
(A) Local recurrence-free survival and (B) radionecrosis-free survival CPB: conformal partial brain; SRT: stereotactic radiosurgery

Twenty-six patients received CPB following the resection of brain metastases with a mean follow-up of 18.2 months. Two of these patients underwent adjuvant external beam radiation postresection for two separate brain metastases, resulting in 28 distinct treatment instances. Three patients were excluded due to the absence of follow-up imaging resulting in 25 evaluable treatments. The average interval between resection and the completion of radiation was 55 days, and patients received a median dose of 3300 Gy over 11 fractions prescribed to the 98% line (Table 3). Fifteen cavities were treated using a 3-D technique and 10 were treated using IMRT. None of the patients experienced local tumor bed recurrence or radiation necrosis (Figure 3). However, 19 patients experienced distant brain recurrences, with a median time of 7.4 months (Table 4). Of these, seven developed nodular subtype leptomeningeal disease.

**Table 3:** Comparison of treatment parameters between CPB and SRT treatment groups CPB: Conformal partial brain; SRT: stereotactic radiosurgery

Characteristic	CPB N=25	SRT N=32
Total dose, mean (SD)	3298 (10)	2578.13 (356.94)
Number of fractions, mean (SD)	10.96 (0.2)	3.28 (1.05)
Prescription isodose line, mean (SD)	98 (2.51)	80.43 (2.91)

**Table 4:** Comparison of clinical outcomes between CPB and SRT treatment groups *p<0.05; **p<0.001 t-test for difference in means: follow-up, time to distant recurrence, overall survival; t-test for difference in proportions: local recurrence, distant recurrence, radiation necrosis, death CPB: conformal partial brain; SRT: stereotactic radiosurgery

Characteristic	CPB N=25	SRT N=32	p-value
Follow-up (months), mean (range)	18.2 (1.0, 50.0)	21.3 (1.0, 60.0)	0.502
Local recurrence, n (%)	0 (0.0%)	7 (21.9%)	0.0133*
Distal recurrence, n (%)	20 (80.0%)	11 (34.38%)	0.001**
Radiation necrosis, n (%)	0 (0.0%)	7 (21.9%)	0.0133*
Death, n (%)	15 (60.0%)	11 (34.4%)	0.056
Time to local recurrence (months), mean (range)	N/A	7.87 (1.22, 21.01)	N/A
Time to distal recurrence (months), mean (range)	7.36 (0.73, 27.29)	7.95 (0.65, 23.40)	0.826
Time to necrosis (months), mean (range)	N/A	21.08 (4.35, 51.56)	N/A
Overall survival (months), mean (range)	13.55 (0.96, 41.03)	15.17 (0.67, 41.63)	0.772

Stereotactic Radiation Therapy

Thirty-five patients underwent radiosurgery following brain metastasis resection with a mean follow-up of 21.3 months. One of these patients received adjuvant radiosurgery radiation postresection for two different brain metastases, resulting in 36 distinct treatment instances. Four patients were excluded due to the lack of follow-up imaging resulting in 32 evaluable treatments. On average, there was a 56-day interval between resection and radiation, and the postoperative tumor beds were prescribed a median dose of 24 Gy in 3 fractions to the 80% isodose line. The average dose was 25.8 Gy in 3.3 fractions (Table 3). Seven patients experienced local recurrence within the tumor bed, with a mean time to recurrence of 7.87 months. Radiation necrosis developed in seven patients (Figure 3). Distant brain recurrence occurred in 12 patients, with a median time of 7.95 months (Table 4). There were no cases of leptomeningeal recurrence.

Toxicity

Acute and late toxicities to the central nervous system (CNS) were classified using the Radiation Therapy Oncology Group (RTOG) criteria [12]. Most patients experienced only grade 1 RTOG acute toxicities such as alopecia, mild headache, and fatigue. In the CPB arm, there were no cases of late radionecrosis. In contrast, 7 out of 32 patients in the SRT arm experienced radionecrosis at the treatment site. There was one RTOG grade four late complication of RN-induced status epilepticus.

## Discussion

One standard of care after resection of a single brain metastasis in patients with limited intracranial disease is to treat the cavity with SRT to minimize the risk of recurrence. The 2022 ASTRO/ASCO/SNO guidelines recommend stereotactic radiation in one to five fractions [10]. SRT is highly conformal, effectively sparing normal brain tissue while delivering a high BED to the brain targets. The seminal NCCTG N107C randomized trial demonstrated that radiosurgery to the tumor bed resulted in better cognitive deterioration-free survival compared to WBRT. Nevertheless, the one-year LC in this study was only 40.7%, compared to 81.5% in the WBRT arm [9]. The SRS control rate reported in the NRG study was considerably inferior to the 81% LC rate reported in meta-analyses of retrospective studies [13]. The study did not report the radiation necrosis rate, suggesting the possibility that the high local failure rate may have included both local recurrences and cases of radiation necrosis. An ongoing NRG study is examining SRT to the tumor bed to minimize the risk of radiation necrosis (NCT04114981) [14].

While SRT is effective for treating well-defined targets such as metastases, accurately identifying the volume at risk can be challenging due to the difficulty in accurately delineating the cavity and its tendency to contract over time [15]. Salkeld et al. showed that measurable changes can occur as quickly as seven days after the acquisition of a planning MRI, which could necessitate changes in management in as many as 41% of patients [16]. Additionally, surgical resection may cause subclinical tumor seeding along the tumor bed and surgical tract. The combination of a dynamic cavity volume and potential subclinical seeding increases the difficulty of accurately contouring the volume at risk [17]. The highly conformal SRT dose distribution presents a dilemma, in that conservative margins can increase the risk of local recurrence, whereas generous margins increase the risk of radiation necrosis [18]. We hypothesized that the CPB would be a more effective option for resected brain metastases due to more generous margins around the cavity, surgical tract, and dural surface which could decrease the risk of a geographic miss.

In this study, we retrospectively compared two regimens: a one- to five-fraction SRT technique and an 11-fraction CPB technique. Despite having much larger cavity sizes, the CPB regimen was associated with a significantly reduced risk of both local recurrence and radiation necrosis. None of the patients in the CPB cohort experienced local recurrence or radiation necrosis, whereas in the SRT cohort, 21.9% of patients experienced local recurrence and 21.9% had radiographic radiation necrosis. Most of the radiation necrosis cases were asymptomatic, although it did induce considerable patient anxiety and the need for further workups, such as MR perfusion scans, to rule out recurrence. One patient had symptomatic radiation necrosis, which resulted in an RTOG grade four toxicity of status epilepticus. There was a statistically higher incidence of distant brain failure and leptomeningeal seeding in the CPB arm versus the SRT arm. About 80% of the CPB arm patients had distant brain recurrence versus 35% of the SRT patients. This difference was not significant in multivariate analysis. Specifically, the CPB cohort experienced seven instances of nodular leptomeningeal disease, while the SRT arm did not experience any leptomeningeal disease. This discrepancy was likely due to the much larger cavity sizes in the CPB arm, which is associated with subclinical seeding of the CSF and, consequently, a higher leptomeningeal disease rate [19]. The mean cavity size in the CPB arm was 73 ccs, compared to 13 ccs in the SRT arm. Metastases in the infratentorial location and postoperative hemorrhage, more common in the CPB cohort, may have also contributed to the higher rates of leptomeningeal disease [20,21]. A local postoperative radiation technique would be unlikely to impact the risk of distant brain relapse. Preoperative SRT is a promising technique currently being studied in an NRG randomized trial to determine whether the risk of nodular leptomeningeal disease can be decreased (BN-012) [22].

The first retrospective report on CPB with a 3-D external beam technique to the metastatic tumor bed was published by Byrne et al. in 2020. Forty-five patients received CPB radiation doses ranging from 30 Gy to 39 Gy over 10 to 13 fractions at 3 Gy per fraction. The one-year LC rate was 88.2%, with no cases of radiation necrosis (RN) [23]. To our knowledge, our study is the first confirmatory study regarding the use of adjuvant partial brain external beam radiation therapy with moderate dose escalation to the tumor cavity; it is also the first to compare SRT and CPB in the postoperative brain metastasis setting. A strength of this study is that treatments were administered in a single department of a large community hospital with a relatively uniform approach; target volumes were reviewed by at least one other radiation oncologist in our department.

Most of the CPB patients were treated using a 3-D planning technique. This technique is simple and inexpensive and can be performed without specialized equipment, making it ideal for low-resource settings [24]. For more complex target volumes or to spare critical normal structures such as the brainstem or optic chiasm, we used IMRT. The current paradigm in many centers is SRS to the cavity for small cavity sizes and otherwise WBRT [25]. CPB represents a conformal treatment option for these patients with higher risk and large cavity sizes that has the advantage of avoiding the permanent neurocognitive effects of WBRT.

Taken together, the two published series of the CPB technique [19,23] support improved LC and decreased late toxicity versus the standard of care SRT technique to the tumor bed. It is well known that the risk of radiation necrosis increases as the target volume increases, and CPB may be especially suitable for large cavities or when it is difficult to delineate the tumor bed. Initially, patients were treated with either SRT or CPB based on physician preference. Over time, high-risk (i.e., postoperative hemorrhage or poorly defined cavities) and large surgical bed patients were preferentially treated in our department with the CPB technique, potentially reducing the rates of local failure and radiation necrosis in this cohort.

Limitations

This study's retrospective design introduces limitations, including the lack of randomization among patient groups, which resulted in differences in the average lesion size between the groups. Additionally, longer follow-up periods are needed to establish consistent patterns in radiation therapy outcomes over several years. These challenges are further compounded by the relatively small sample size of the study cohorts, which may affect the generalizability of the findings.

## Conclusions

In this single institution series, adjuvant CPB irradiation of the metastatic tumor bed was statistically associated with a lower risk of both local failure and radiation necrosis compared to standard-of-care adjuvant SRT. Despite the cavity volumes being much larger, none of the CPB patients suffered either local failure or radiation necrosis. Postoperative CPB irradiation with 33 Gy in 11 fractions to the metastatic tumor bed may represent a safer and more effective method than SRT for large cavity sizes or when it is difficult to delineate the tumor bed. Ideally, a prospective randomized trial would be performed to compare CPB radiation therapy to the standard SRT technique after surgical resection of brain metastases.
